# MR imaging in multiple system atrophy: Its role in “splitting” parkinsonism

**DOI:** 10.4103/0972-2327.44565

**Published:** 2008

**Authors:** J. Vijayan, S. Sinha, S. Ravishankar, A. B. Taly

**Affiliations:** Department of Neurology, National Institute of Mental Health and Neurosciences, Bangalore, India; 1 Department of Neuroradiology, National Institute of Mental Health and Neurosciences, Bangalore, India

We report the case of a 59-year-old woman with Multiple System Atrophy – cerebellar (MSA-C) type, who presented with two years’ history of gait unsteadiness associated with dysarthria and bladder incontinence of one-year duration. There was no family history of similar illness. She had hypomimia, with a reduced blink rate. The mini-mental score (Folstein) was 29/30. The patient's eye movement revealed slow dysmetric saccades and broken pursuits. Her speech was slow, hypophonic and monotonous. There was cogwheel rigidity of right > left upper extremity. Intention tremor, dyssynergia and dysmetria, and dysdiadokinesia were noted. The muscle stretch reflexes were brisk and her plantar response was flexor. The gait was broad-based, with variable stride length, poor foot clearance, sway towards the right, with reduced arm swing, and start and turn hesitation. Autonomic function test revealed significant postural hypotension (20 mmHg) on tilt table test; mild detrusor over activity with sphincter dyssynergia, significant residual urine and reduced bladder capacity on urodynamic assessment. There was no improvement with levo-dopa. A possibility of MSA-c was considered in view of prominent cerebellar signs, Parkinsonism and dysautonomia.

An MRI of the brain revealed mild to moderate cortical atrophy, with disproportionate atrophy of pons and cerebellum. T2/ PD/FLAIR sequences revealed the classical ‘hot-cross bun’ sign in the pons characterized by cruciate hyperintensity secondary to atrophy of the transverse pontine fibers. There was hyperintensity of bilateral middle cerebellar peduncle (MCP) - the bright ‘MCP sign’ and putaminal atrophy. There was no restricted diffusion of the signal changes on apparent diffusion coefficient (ADC) mapping. Serial MR imaging after a two-year period revealed increase in atrophy and decrease in signal changes of MCP. The ‘hot cross bun’ sign was better appreciated.

Our patient had classically described imaging findings associated with MSA-C viz pontocerebellar atrophy, bright middle cerebellar peduncles, ‘hot cross bun’ sign, and putaminal atrophy. There was, however, no signal changes involving the putamen i.e. ‘bright putaminal sign’ or restricted diffusion on ADC. Signal alterations involving the pontocerebellar region, especially the middle cerebellar peduncles would suggest MSA-C.[1] These findings are useful in differentiating MSA-C from the other types of sporadic cerebellar ataxia, with prominent extracerebellar features.[2]

The above observations with putaminal signals alterations suggest extrapyramidal involvement, which are characteristic of MSA.[3] In a study by Schrag *et al*., the sensitivity of MRI for revealing any of the above mentioned abnormalities was 87.5% and the specificity to differentiate between MSA and idiopathic Parkinson's disease was 93.3%, and to differentiate between MSA and controls was 90.6%.[4] Putaminal abnormalities had a sensitivity of 50%, with positive predictive value of 82%.[4] Lee *et al*. showed that patients with predominant cerebellar symptoms had signal changes in MCP and that there was a direct one-to-one relationship between the severity of signal changes and the cerebellar symptoms. This was, however, not evident in patients with dominant Parkinsonism and hyperintense putaminal sign.[5] Bhattacharya *et al*. had also pointed out the usefulness of imaging studies in differentiating the various types of MSAs and suggested an algorithm for diagnosis.[6] A recent study[7] too has shown the usefulness of diffusion weighted MRI in differentiating the various types of Parkinsonism, with restricted ADC of the middle cerebellar peduncle, suggesting multiple system atrophy. There have been a few studies looking at follow-up imaging characteristics, which have shown a positive correlation with the rate and degree of middle cerebellar atrophy to Parkinsonism of MSA type; and this regional atrophy is a better discriminating factor.[8] This case reiterates the importance of MRI in diagnosing and differentiating the various types of Multiple System Atrophy. There is limited diagnostic role of serial imaging in MSA, since it did not significantly add to the findings noted in the initial study.

**Figure 1 (A-D):**
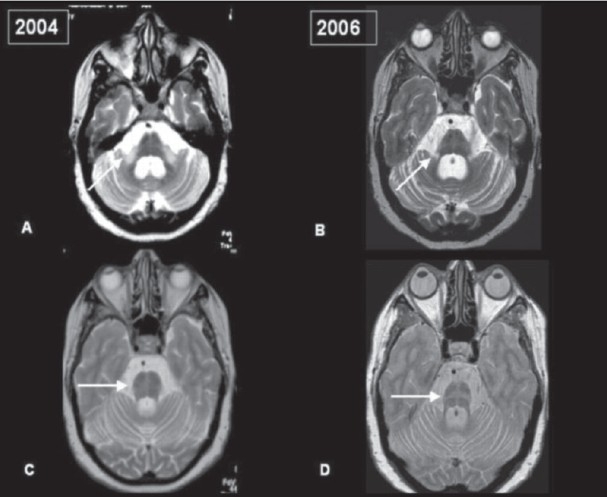
Serial MRI of brain of a patient with MSA-c over a 2-year period. A, B: T2W axial sequences showing hyperintensity of bilateral middle cerebellar peduncle. The repeat image after two years (B) revealed mild increase of brainstem and cerebellar atrophy and decrease in signal change. ‘Hot cross bun’ sign is also evident. C, D: PD axial images revealing the characteristic ‘hot cross bun’ sign in pons (2004) and after two years (2006)
